# Therapy to target renal cell carcinoma using ^131^I-labeled B7-H3 monoclonal antibody

**DOI:** 10.18632/oncotarget.8550

**Published:** 2016-04-02

**Authors:** Gongcheng Wang, Ziyu Wu, Yunyan Wang, Xueqin Li, Guangbo Zhang, Jianquan Hou

**Affiliations:** ^1^ Department of Urology, Huai’an First People's Hospital, Nanjing Medical University, Huai’an, Jiangsu, China; ^2^ Department of Urology, Huai’an Hospital Affiliated of Xuzhou Medical College and Huai’an Second People's Hospital, Huai’an, Jiangsu, China; ^3^ Department of Gerontology, Huai’an First People's Hospital, Nanjing Medical University, Huai’an, Jiangsu, China; ^4^ Department of Clinical Immunology Laboratory, The First Affiliated Hospital of Soochow University, Suzhou, Jiangsu, China; ^5^ Department of Urology, The First Affiliated Hospital of Soochow University, Suzhou, Jiangsu, China

**Keywords:** B7-H3, mice, nude, renal cell carcinoma, targeting therapy, monoclonal antibody

## Abstract

B7-H3 is a tumor-associated antigen that plays a critical role in potential tumor-targeted therapy. In this study, we aimed to assess the radiobiological effect of ^131^I-labeled B7-H3 monoclonal antibody (^131^I-4H7) in nude mice with human renal cell carcinoma (RCC) and evaluate the effect of ^131^I-4H7 on RCC treatment. The radiobiological activity and tumor uptake of ^131^I-4H7, and its effect on tumor growth were measured. ^131^I-4H7 was absorbed by the tumor and reached its maximal uptake rate (3.32% injected dose [ID]/g) at 24 h, at which point the drug concentration in the tumor was 7.36-, 2.06-, 1.80-, and 2.78-fold higher than that in muscle, kidneys, liver, and heart, respectively. Measurements and positron emission tomography–computed tomography imaging showed that tumor development was significantly inhibited by ^131^I-4H7. HE staining revealed that ^131^I-4H7 significantly injures tumor cells. Our results suggest that ^131^I-4H7 is markedly absorbed by the tumor and did suppress the development of RCC xenografted tumors in nude mice, which might provide a new candidate for antibody-mediated targeted radiotherapy in human RCC.

## INTRODUCTION

Renal cell carcinoma (RCC) is the most common malignant tumor in the adult kidney, accounting for 2.0-3.0% of all human malignancies [1], and its occurrence has increased in recent years [2–4]. The incidence of RCC worldwide is approximately 209, 000 new cases per year and 102, 000 deaths per year [5]. In the United States, it is estimated that there will be ~61, 560 new cases and 14, 080 deaths of kidney and renal pelvis cancer in 2015 [6]. Recent studies have clearly shown that the clear-cell tumor subtype is the most frequent, accounting for >80% of all RCCs [7, 8]. Radical surgery is the most effective treatment option for early stage RCC. Unfortunately, 15-25% of patients present with a metastatic disease at the time of diagnosis and 30% of the localized disease will relapse into a metastatic setting during the years following surgery. The lack of any demonstrable efficacy from chemotherapy and radiation therapy in advanced RCC has led to a 5.0-year survival rate ranging from 5.0 to 10.0% [9, 10].

B7-H3, a member of the B7 immunoregulatory family, was identified in 2001 by database searches of a human dendritic cell-derived cDNA library [11]. B7-H3 is broadly expressed at the transcriptional level and is found in a wide spectrum of both human solid tumors and normal tissues, while at the protein level, B7-H3 can be expressed in dendritic cells and in the liver, lungs, and prostate as well as in most tumor cell lines. B7-H3 was identified as the most differentially expressed cell-surface tumor-specific endothelial marker [12]. Studies have shown that tumor B7-H3 expression is correlated with poor patient survival in both clear cell RCC (ccRCC) and urothelial cell carcinoma [13, 14]. Qin et al. [15]. found that the expression of B7-H3 was not only detected in ccRCC specimens, but was also confirmed in RCC vasculature, and that the vascular B7-H3 expression was associated with multiple adverse clinical and pathologic features. In addition, B7-H3 expression in tumor vasculature was also correlated with poor survival, suggesting that this ligand might play an important role in tumor cell migration [15, 16]; therefore, B7-H3 might be a useful target for tumor-specific antiangiogenic therapies [17].

Targeted therapy, which has radically altered the treatment of late-stage RCC in recent years, relies on the following two main groups of agents: vascular endothelial growth factor (VEGF)-targeting drugs and mammalian target of rapamycin (mTOR) inhibitors. Nevertheless, despite recent success, complete response to antiangiogenic therapies is rare. One study showed that 80.95% of patients receiving ^131^I-B7-H3 monoclonal antibody (8H9) salvage therapy had prolonged survival times after a central nervous system (CNS) neuroblastoma relapse [18]. These findings led us to hypothesize that the specific expression of B7-H3 in the RCC vasculature system could make it a prominent carrier for B7-H3 image- and receptor-mediated targeted radiotherapy. Utilizing ^131^I as a radiotherapeutic nuclide and monoclonal antibody B7-H3 (4H7) as a carrier, the feasibility and efficacy of B7-H3-mediated ^131^I-4H7 targeted radiotherapy was analyzed in a human RCC xenograft model.

## RESULTS

### Clinical tissue samples and immunohistochemical staining

**Figure 1:**
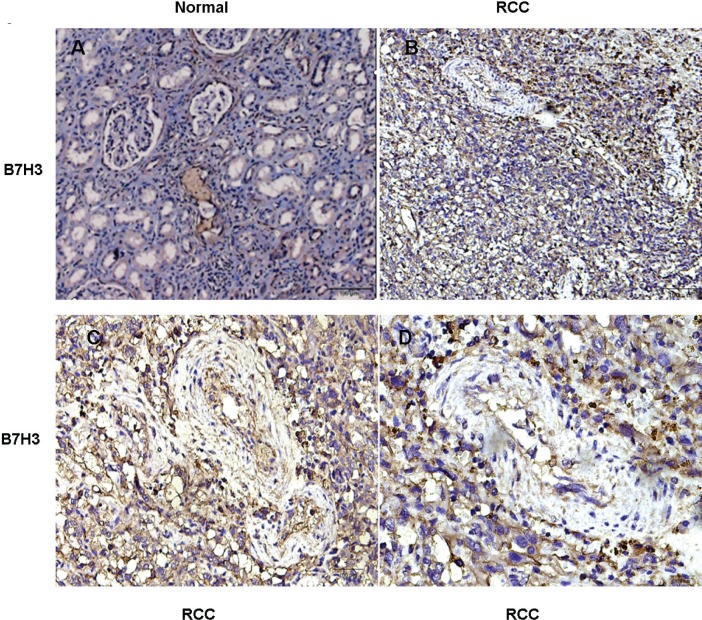
Immunohistochemical staining for B7-H3 in clinical specimens **A.** In normal renal tissue, renal tubules were positive staining for B7-H3 and the stroma were negative; **B.**-**D.** In RCC tissue, B7-H3 was positive staining extensively, including the tumor cells, vasculature, and the stroma. Representative images are shown (*n* = 8) (A: ×100, B: ×100, C: ×200, D: ×400).

Immunohistochemical (IHC) staining analysis showed significantly more B7-H3 staining in the tumor cells, tumor vasculatures, and stroma of the ccRCC samples than in normal renal specimens (Figure 1A-1D), which was consistent with the previously reported study results [16].

### Chemistry and radiochemistry

**Figure 2:**
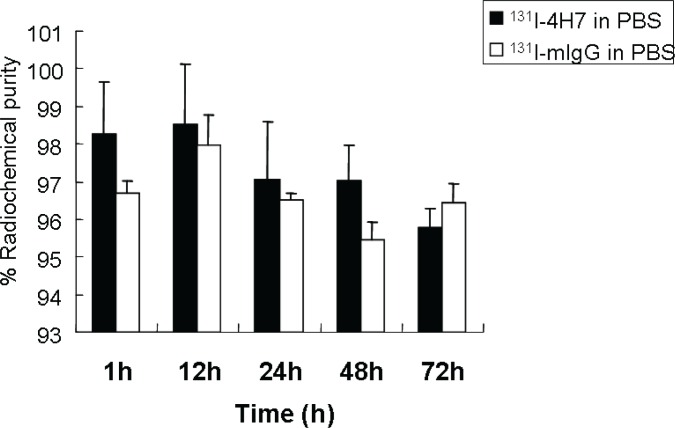
*In vitro* stability of ^131^I-4H7 and ^131^I-mIgG in phosphate buffered saline (pH 7.4) at 37°C for 1.0, 12, 24, 48, and 72 h Their radiochemical purities were >95% and >95% of ^131^I-4H7 and ^131^I-mIgG remained intact in PBS after 72 h of incubation.

^131^I-4H7 and ^131^I-mIgG were prepared and the radiochemical purities of both were >95%. The *in vitro* stability of ^131^I-4H7 in PBS (pH 7.4) at 37°C is shown in Figure 2. After 72 h of incubation, >95% of the ^131^I-4H7 and ^131^I-mIgG remained intact in PBS.

### Biodistribution studies

**Figure 3:**
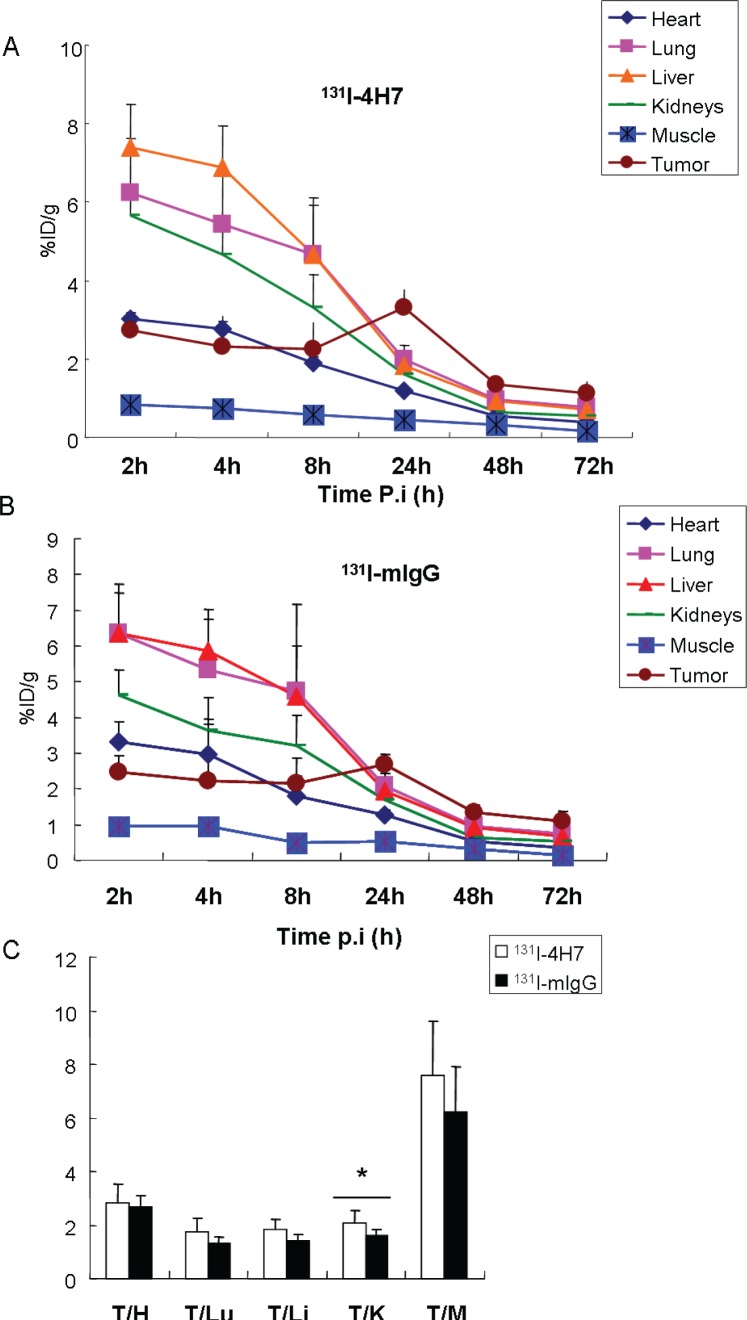
Biodistribution of **A.** ^131^I-4H7 and **B.** ^131^I-mIgG in 786-0 tumor, heart, lung, liver, kidneys, and muscle after intravenous injection of 3.7 MBq ^131^I-4H7 or ^131^I-mIgG. **C.** Ratio of tumor to major organs (heart, lung, liver, kidneys, and muscle) based on the biodistribution data at 24-h post injection. Error bar was calculated as the standard deviation (*n* = 6, mean ± SD).

Tissue distribution data for ^131^I-4H7 and ^131^I-mIgG in tumor-bearing nude mice are given as the percentage of administered activity per gram of tissue (%ID/g) (Figure 3). *In vivo* biodistribution of injected ^131^I-4H7 and ^131^I-mIgG was examined in these mice.

For ^131^I-4H7, the tumor uptake was determined to be 2.72 ± 0.49, 2.32 ± 0.77, 2.25 ± 0.69, 3.32 ± 0.46, 1.34 ± 0.20, and 1.13 ± 0.28% ID/g at 2.0, 4.0, 8.0, 24, 48, and 72 h, respectively. Its uptake rate peaked at 24 h, at which point the drug concentration in the tumor was 7.36-, 2.06-, 1.80-, 1.67-, and 2.78-fold higher than that in the muscle, kidneys, liver, lung and heart, respectively (Figure 3A). For ^131^I-mIgG, the tumor uptake was 2.46 ± 0.48, 2.21 ± 1.73, 2.15 ± 0.69, 2.67 ± 0.29, 1.33 ± 0.20, and 1.11 ± 0.28% ID/g at 2.0, 4.0, 8.0, 24, 48, and 72 h, respectively. Its uptake rate also peaked at 24 h, at which point the drug concentration in the tumor was 5.02-, 1.58-, 1.39-, 1.29-, and 2.09-fold higher than that in muscle, kidneys, liver, lung and heart, respectively (Figure 3B). ^131^I-4H7 exhibited 7.39 ± 1.11% ID/g liver uptake compared with 6.36 ± 1.11% ID/g in ^131^I-mIgG at 2.0 h post-injection (Figure 3A-3B). ^131^I-4H7 showed 5.67 ± 0.68% ID/g of kidney uptake, which is higher than that of ^131^I-mIgG (4.64 ± 0.68% ID/g) at 2.0 h pi (Figure 3A and 3B). It might be the reason that 4H7 metabolized mainly through the liver and kidneys. The nonspecific uptake in the muscle was at a very low level for both tracers. ^131^I-4H7 exhibited greater tumor uptake at the early time point and better tumor retention, indicating a longer circulation time. In addition, ^131^I-4H7 showed greater tumor uptake compared to that of ^131^I-mIgG, and the ^131^I-4H7 tumor/kidney ratio of was significantly higher than that of ^131^I-mIgG (Figure 3C). Similar tumor/muscle, tumor/liver, and tumor/heart ratios were observed for both ^131^I-4H7 and ^131^I-mIgG (Figure 3C).

### Positron emission tomography /computed tomography (PET-CT) imaging studies

**Figure 4:**
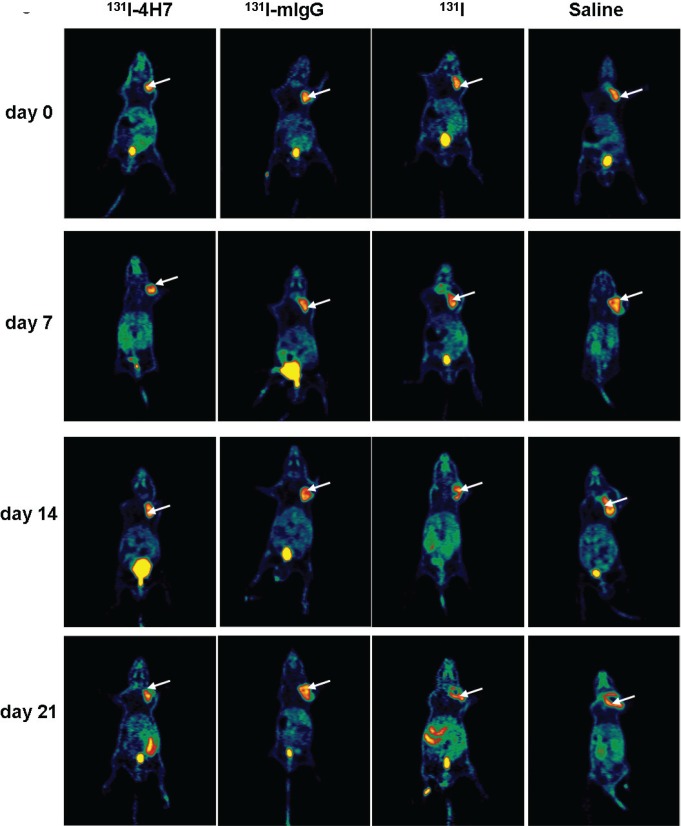
Representative decay-corrected whole-body PET-CT images of the effect of drugs on tumor growth Nude mice with renal cell carcinoma (RCC) xenografts were injected with saline, ^131^I, ^131^I-mIgG, or ^131^I-4H7. Tumor growth was monitored on days 0, 7.0, 14, and 21. Representative images are shown (*n* = 4).

The effect of ^131^I-4H7, ^131^I-mIgG, ^131^I, and saline on tumor xenograft growth in nude mice was evaluated by static PET-CT at different time points after intravenous injection. Rapid growth was observed in the groups treated with saline and ^131^I, in contrast, slow growth was observed in mice treated with ^131^I-4H7 and ^131^I-mIgG. Group treated with ^131^I-4H7 grew more slowly than those treated with ^131^I-mIgG (Figure 4 and Supplementary Table 1).

### Effect of ^131^I-4H7 on tumor xenograft growth

**Figure 5:**
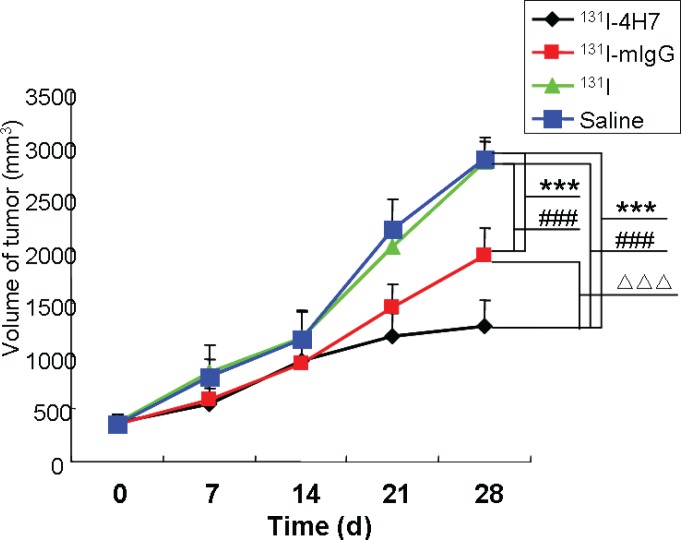
The effect of drugs on tumor growth Nude mice with renal cell carcinoma (RCC) xenografts were injected with saline, ^131^I, ^131^I-mIgG, or ^131^I-4H7. Tumor growth was monitored over 28 d. Data are mean ± SD, *n* = 8 (^***^: *p* < 0.001, ^131^I, ^131^I-mIgG, and ^131^I-4H7 group vs. Saline group. ^###^: *p* < 0.001, ^131^I-mIgG, and ^131^I-4H7 group vs. ^131^I group. ^ΔΔΔ^: *p* < 0.001, ^131^I-4H7 group vs. ^131^I-mIgG group).

The effect of ^131^I-4H7, ^131^I-mIgG, ^131^I, and saline on tumor xenograft growth in nude mice is shown in Figure 5 (and Supplementary Table 2). Rapid growth was observed in the group treated with saline and ^131^I; the tumors were 2.23, 3.21, 6.09, and 7.94 times and 2.30, 3.21, 5.55, and 7.77 times the size of the original at days 7.0, 14, 21, and 28, respectively. The growth curves of ^131^I-4H7 and ^131^I-mIgG groups were also different, with tumors reaching 1.46 to 1.66, 2.54 to 2.62, 3.18 to 4.15, and 3.44 to 5.53 times the original size, respectively, at days 7.0, 14, 21, and 28.

A paired Student's *t* test revealed a significant difference between the group treated with saline and the groups treated with ^131^I-4H7 and ^131^I-mIgG at day 28, while there was no difference between the saline group and ^131^I group. This shows that treatment with ^131^I-4H7 and ^131^I-mIgG significantly inhibited tumor growth and changed the original growth rate of the xenografted tumors, and that the inhibiting effect was more significant in the ^131^I-4H7 group. At day 28, the group treated with ^131^I-4H7 showed a 55.19% inhibition of tumor growth (Supplementary Table 3). There was also a significant difference between the group injected with ^131^I-4H7 and that injected with ^131^I-mIgG (31.92%) (Supplementary Table 3), which suggested that ^131^I-4H7 acted more significantly to suppress tumor growth than ^131^I-mIgG.

### Hematoxylin-eosin staining

**Figure 6:**
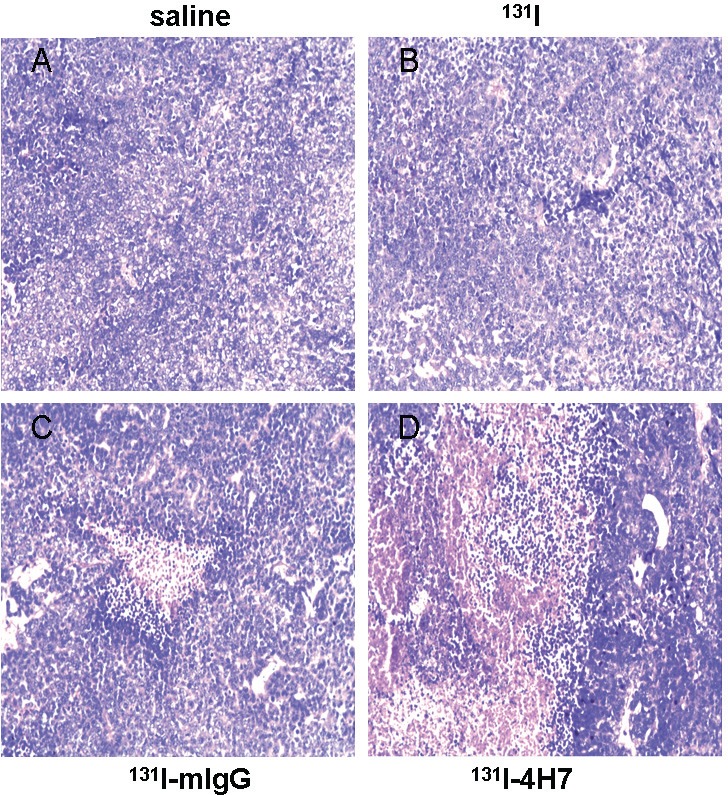
Samples dissected from renal cell carcinoma (RCC) cells treated with ^131^I-4H7, ^131^I-mIgG, ^131^I, or saline were sectioned and stained with hematoxylin-eosin for visualization of general morphology Representative images are shown (*n* = 8). Cells were treated with **A.** saline, **B.** ^131^I, **C.** ^131^I-mIgG, and **D.** ^131^I-4H7.

The saline- and ^131^I-treated groups were characterized by well-developed proliferations of ccRCC cells, with a compact arrangement and diffusely distributed invading cancer nests; the nuclei were of various sizes and there was no apparent necrosis in the cancer tissue (Figure 6A-6B). The ^131^I-4H7 and ^131^I-mIgG treated groups were characterized by a large field of ccRCC cell necrosis, with dying tumor cells undergoing vacuolar degeneration of the cytoplasm (Figure 6C-6D). There were more regions of necrosis in the ^131^I-4H7 group than in the ^131^I-mIgG group (Figure 6C-6D).

## DISCUSSION

RCC is the most common malignant tumor in the adult kidney and its incidence increases annually. RCC is a heterogeneous group of cancers with disparate genetic and molecular alterations underlying the various recognized histological subtypes. Considering that RCC possesses multiple drug-resistant genes and is not sensitive to traditional chemotherapy and radiotherapy, prognosis of advanced RCC remains poor, with <10% of patients still living 5.0 years after diagnosis of the metastatic disease [9, 10]. Monoclonal antibodies are often directed toward cell-surface molecules and can be radiolabeled for the imaging or treatment of cancer and other ailments [19–21]. Targeted agents against VEGF, VEGFR, or mTOR continue to play a crucial role in the management of metastatic ccRCC [22].

B7-H3 was discovered firstly by Chen in 2001 [11]; however, the physiological and pathological role of B7-H3 is far less well known and both stimulatory and inhibitory effects have been described for this ligand. Recent studies have indicated that B7-H3 overexpression was found in several tumors, such as prostate cancer, non-small-cell lung carcinoma, and RCC. In addition, B7-H3 is expressed in both RCC cells and tumor vessels and was marked as expressed in RCC vasculatures in some studies as high as 98% [15], which indicates that B7-H3 could act as a potent new cancer vessel-specific carrier to selectively deliver antiangiogenic or vascular-disrupting agents to diseased tissues, minimize any potential side effects, and could help predict the clinical outcome of using different targeted agents in the treatment of RCC.

In the present study, the level of B7-H3 expression was higher in the tumor cells, tumor vasculatures, and stroma of the ccRCC samples than in normal renal specimens, which was consistent with previous studies, providing a theoretical basis for ^131^I-labeled targeted radiotherapy for RCC using B7-H3 monoclonal antibody (4H7) as a carrier. The biodistribution studies indicated that the radiochemical purities of both ^131^I-4H7 and ^131^I-mIgG were >95%, and the stability of both traces in PBS was >95% after incubating for 72 h. Moreover, the tissue distribution experiments showed that ^131^I-4H7 was markedly absorbed by tumor tissue in tumor-bearing nude mice, reaching a maximal uptake rate (3.32% ID/g) at 24 h after intravenous injection. The retention of ^131^I-4H7 was such that at 72 h, the uptake rate remained at 1.13% ID/g, indicating that 4H7 kept its activity after being radioiodinated by ^131^I, which showed that it could be absorbed by RCC tissues and suggested that it might make a useful clinical candidate for a RCC-targeting treatment option. All normal tissue concentrations decreased over time but this did not occur to a great extent in the tumors. It might be possible that the uptake rate of ^131^I-4H7 does not distinctly reflect the accumulation of 4H7 *in vivo* because of the many enzymes that can accelerate the catabolism and deiodination of ^131^I-4H7 in normal tissue, but in tumors, this does not appear to be the case, and the accumulation of ^131^I-4H7 is gradually increased by the action of B7-H3. This is advantageous to the treatment of the tumors with radiotherapy.

Our therapeutic experiments showed that ^131^I-4H7 and ^131^I-mIgG treatment through intravenous injection could inhibit tumor growth and alter the ultrastructure and histopathology of RCC cells, resulting in irreversible injury and wide necrosis, and that the therapeutic effect was more pronounced in the ^131^I-4H7 group than in the ^131^I-mIgG group. The results indicated that the targeted effect of ^131^I-4H7 is better than that in ^131^I-mIgG. Moderate energy β-particles from ^131^I-4H7 targeted to tumor vasculatures *in vivo* were sufficiently damaging to tumor vasculatures, which could lead to damaging tumor angiogenesis, causing the hypoxic ischemia necrosis of tumor cells. Several researches have indicated that cancer cell-specific B7-H3 expression in ccRCC is ~20% [13, 15], although lower than that in tumor vasculatures, and that the direct cytotoxic effect on ccRCC cells of ^131^I-4H7 cannot be completely ruled out.

In the therapeutic experiments, ^131^I-4H7 treatment could inhibit tumor growth and cause irreversible injury to RCC cells and tumor vasculatures, but we did not find tumor regression in the ^131^I-4H7 group, which could be related to the dose of ^131^I-4H7, which might not have been sufficient (Supplementary Figure 1). Further studies are needed to clarify the effect of different doses of ^131^I-4H7 on tumor growth. Meanwhile, there was no metastasis in tumor-bearing nude mice treated with ^131^I-4H7 and the other three agents, which indicated that the dose of ^131^I-4H7 was correct and safe.

In conclusion, our results indicate that the radiobiological effect of ^131^I-4H7 is stable and ^131^I-4H7 did suppress the development of human RCC xenografted tumors in nude mice at day 28, which might provide a new candidate for B7-H3 receptor-mediated targeted radiotherapy in human ccRCC.

## MATERIALS AND METHODS

### Clinical sample collection

Twenty-six patients with ccRCC who underwent surgery for radical nephrectomy were included in the study. Paired samples of ccRCCs and adjacent normal tissues were immediately placed on ice, partitioned, and fixed in 10% formalin. Patients were excluded from the study if they had received chemotherapy or radiation therapy before the surgical procedure or had undergone previous renal surgery. ccRCC specimens were confirmed by postoperative pathological diagnosis. Written informed consent was obtained from each patient and this study was approved by the Ethics Committee of The First Affiliated Hospital of Soochow University and Huai’an First People's Hospital of Nanjing Medical University, China.

### Cell culture and animal model

The human ccRCC-derived cell line 786-0 was purchased from the Institute of Cell Research, Chinese Academy of Sciences, Shanghai, China. Cells were cultured in RPMI 1640 medium (Gibco Invitrogen, New York, USA) supplemented with 10% fetal bovine serum (Gibco Invitrogen, New York, USA) at 37°C in an atmosphere of 5.0% CO_2_. One hundred twenty male BALB/c nude mice (5-6 weeks old and weighing 20-24 g) were obtained from the West China Experimental Animal Center of Sichuan University. These nude mice were bred under aseptic-specified pathogen-free (SPF) conditions and kept at a constant humidity and temperature (25-28°C). Animal experiments were performed according to protocols approved by the Animal Care and Use Committee and were in compliance with the Guidelines on Animal Welfare of the China National Committee for Animal Experiments. Cells of 786-0 (2.0 × 10^7^) in 0.2 mL normal sodium were injected subcutaneously into the right axilla region of the mice. When the tumors reached 8.0 mm in diameter (1.0-2.0 weeks after inoculation), the tumor-bearing mice were used for biodistribution and therapeutic studies.

### B7-H3 monoclonal antibody ^131^I labeling

B7-H3 monoclonal antibody (4H7) was constructed and prepared as previously described [23]. Na^131^I (37 TBq/L) was obtained from the Jiangsu Institute of Nuclear Medicine (China). To prepare the mixture, 100 μL 4H7 were dissolved in 150 μL phosphate buffered saline (PBS, pH 7.4) and mixed with 150 μL Na^131^I (approximately 185 MBq). The solution was mixed with 10 μL chloramine-T and vortexed for 60 s, after which the reaction was immediately halted using 100 μL partial glue solution. The product was purified by column (Bio-Gel P-2 resin, 300 × 10 mm) with PBS. The peak ^131^I-4H7 solution was collected and refiltered through a 0.22-μm membrane. Using Xinhua I filter paper developed with 1:1 acetone and saline, a chromatographic assay of radiochemical purity was performed. Using the same procedure described above, 10 μL murine IgG (mIgG, isotype-matched control; Sigma-Aldrich Corp., St. Louis, MO, USA) were also labeled with ^131^I as the negative control. The stability of ^131^I-4H7 and ^131^I-mIgG in PBS was studied at different time points and the percentage of parent tracer was determined by radio-thin layer chromatography.

### Biodistribution studies

The tumor-bearing nude mice were randomly divided into two groups of 36 mice each and 3.7 MBq (0.1 mCi) ^131^I-4H7 and ^131^I-mIgG, respectively, were injected into the tail vein of each. The mice were then sacrificed at 2.0, 4.0, 8.0, 24, 48, and 72 h after injection. The heart, lungs, kidneys, liver, muscle, and tumor were removed, weighed, and assayed for the ^131^I-4H7 and ^131^I-mIgG uptake rate. The uptake rate was determined by the percentage of radioactivity contained within each gram of tissue compared to the total radioactivity injected into the body. The tumor/nontumor tissue radioactive count (T/NT) was then calculated.

### Therapeutic studies

#### Groups and treatments

From 3.0 d before the experiment until its end, 48 nude mice with xenografted tumors (8-10 mm in diameter) were orally administered 1.0% K^131^I to block free ^131^I uptake by the thyroid. During this time, these mice were fed under SPF conditions. According to body weight and tumor size, the animals were divided into four experimental groups of 12 mice each, which were respectively treated with 0.2 mL saline, 0.2 mL ^131^I (7.4 MBq), 0.2 mL ^131^I-4H7 (7.4 MBq), or 0.2 mL ^131^I-mIgG (7.4 MBq) injected into the tail vein of each. There were no fatalities among the treated mice during the study.

#### PET-CT imaging studies

Following treatment with ^131^I-4H7, ^131^I-mIgG, ^131^I, and saline in the four groups (four mice each), 16 tumor-bearing nude mice were imaged in a supine position with a one-head PET-CT equipped with a pinhole collimator under intraperitoneal injection of 25.0 mg/kg sodium pentobarbital. Static PET-CT images were acquired at days 0, 7.0, 14, and 21 among the four groups.

#### Tumor measurements

Following treatment with ^131^I-4H7, ^131^I-mIgG, ^131^I, and saline of the four groups (eight mice each), xenografted tumors were measured for long dimension and short dimension once a day for 4.0 d and then every 3.0 d for 28 d. Tumor volume was calculated as follows:

Volume = 1/2 × long dimension (cm) × (short dimension [cm])^2^

to draw the growth curve and observe tumor growth [20]. All mice were sacrificed at day 28 and the tumors were removed and weighed. We regarded saline group as the control group, while groups of ^131^I-4H7, ^131^I-mIgG, and ^131^I were all treated groups. The following formula was used to calculate the rate of tumor suppression:

Rate of tumor inhibition (100%) = (1 - mean tumor weight of treated group/mean tumor weight of control group) × 100% [24].

### Hematoxylin-eosin staining and immunohistochemical analysis

Clinical specimens and tumor xenograft specimens were fixed in 10% formalin and embedded in paraffin. We cut the paraffin-embedded specimens into sections of 5.0 μm and stained with hematoxylin and eosin (HE) for general morphological observation. Immunohistochemical (IHC) staining was performed using a rabbit-specific horseradish peroxidase/diaminobenzidine detection IHC kit (Abcam, Cambridge, UK) according to the manufacturer's protocol. Tissue sections were stained with anti-B7H3 (Abcam, Cambridge, UK, 1:200 dilution) for 30 min at room temperature, followed by incubation with secondary rabbit-specific HRP-conjugated antibodies for B7-H3 staining. Samples were visualized using a Nikon Eclipse 80i upright microscope (Nikon, Tokyo, Japan).

### Statistical analyses

Quantitative data are expressed as the mean ± SD. Means were compared using a one-way analysis of variance and Student's *t* test. *p* values < 0.05 were considered statistically significant.

### Ethical standards

All procedures performed in studies involving human participants were in accordance with the ethical standards of the research committee of Soochow University and Nanjing Medical University, China. All participants gave written informed consent before participating in the study.

All animal and tissue sample experiments were performed in accordance with the guidelines of the National Institutes of Health and Soochow University with procedures (ID: 2012074) approved by the Institutional Animal Care and Use Committee of the university.

## SUPPLEMENTARY MATERIAL FIGURES AND TABLES


