# Supplementary material for: Therapy to target renal cell carcinoma using 131I-labeled B7-H3 monoclonal antibody
Source: Oncotarget. 2016 Apr 2;7(17):24888–98. doi: 10.18632/oncotarget.8550 (PMC5029751; doi:10.18632/oncotarget.8550)
Supplement: Supplementary file 1 [file oncotarget-07-24888-s001.pdf]

Therapy to target renal cell carcinoma using ^{131}I-labeled B7-H3 monoclonal antibody

Supplementary Material

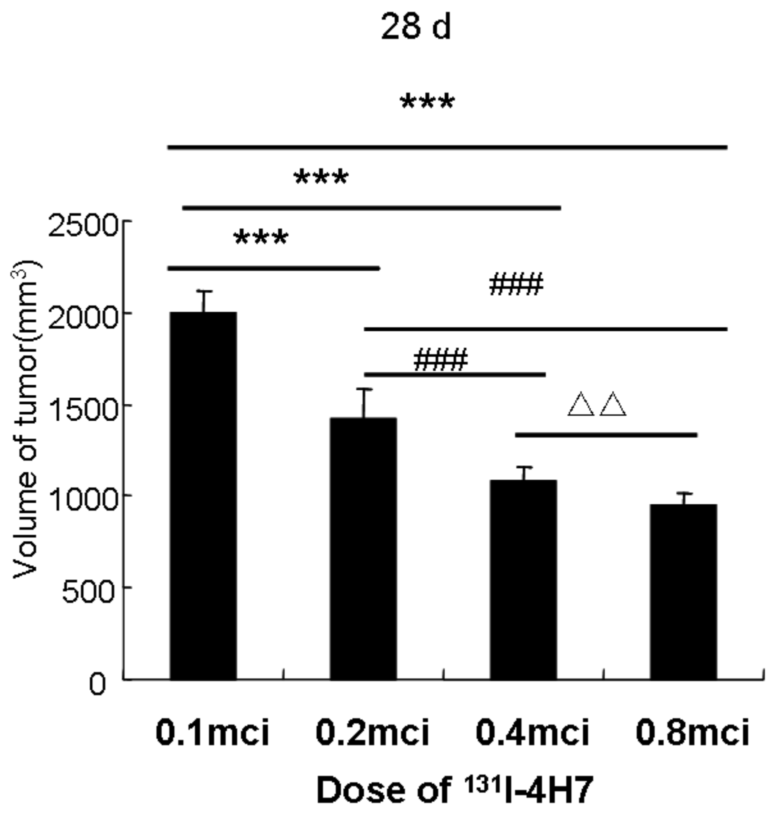

Supplementary Figure 1. Different doses of ^{131}I-4H7 on tumor inhibition. The effect of ^{131}I-4H7 on tumor inhibition was dose dependent, the higher dose of ^{131}I-4H7, the more significant effect of anti-tumor. Data are mean ± SD, n = 8 (***: $p < 0.001$, 0.1 mci group vs. 0.2 mci, 0.4 mci, and 0.8 mci group. $^{\#\#\#}$: $p < 0.001$, 0.2 mci group vs. 0.4 mci and 0.8 mci group. $^{\triangle\triangle}$: $p < 0.01$, 0.4 mci group vs. 0.8 mci group).

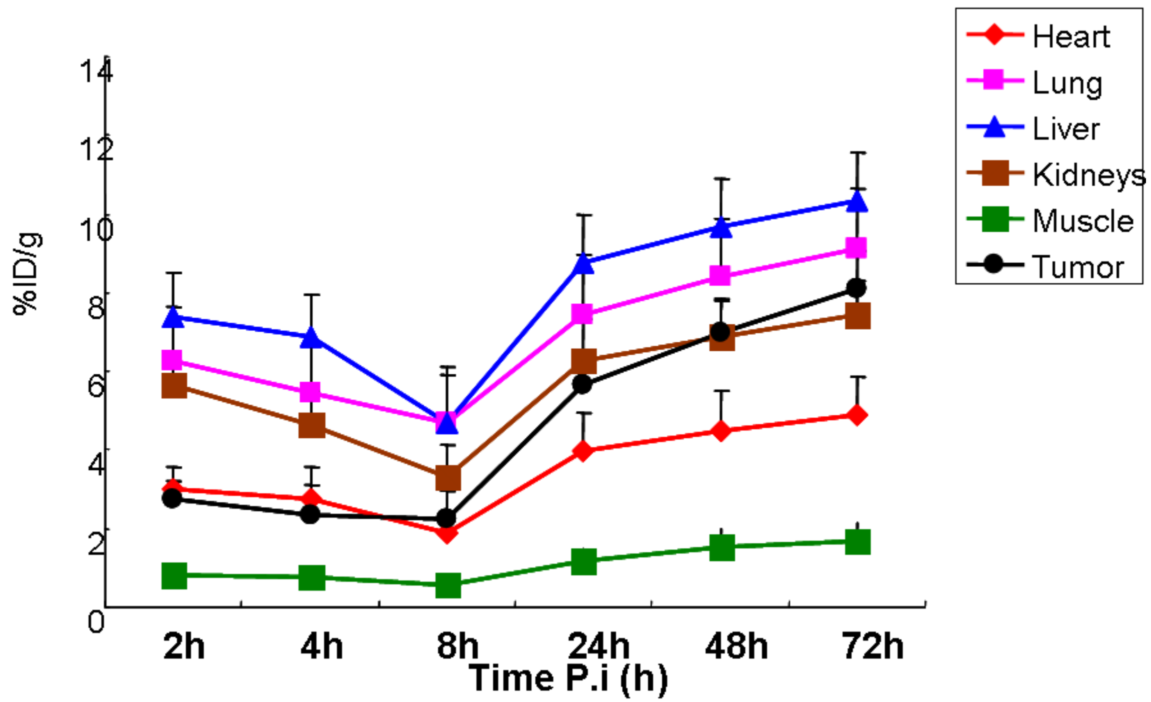

Supplementary Figure 2. Biodistribution of repeated injection of ^{131}I-4H7 (3.7 MBq) in 786-0 tumor, heart, lung, liver, kidneys, and muscle after first intravenous injection of 3.7 MBq ^{131}I-4H7 at 24 h. Data are mean ± SD, n = 6.

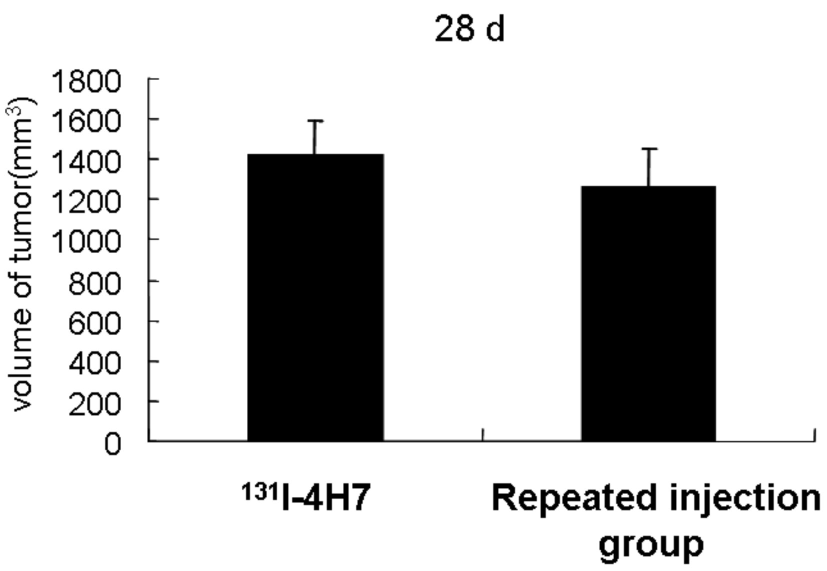

Supplementary Figure 3. The effect of ^{131}I-4H7 and repeated ^{131}I-4H7 on tumor growth. Nude mice with renal cell carcinoma (RCC) xenografts were injected with ^{131}I-4H7 (7.4 MBq) or repeated ^{131}I-4H7 (7.4 MBq) at 24 h after first injection. Tumor growth was monitored over 28 d. Data are mean ± SD, n = 8.

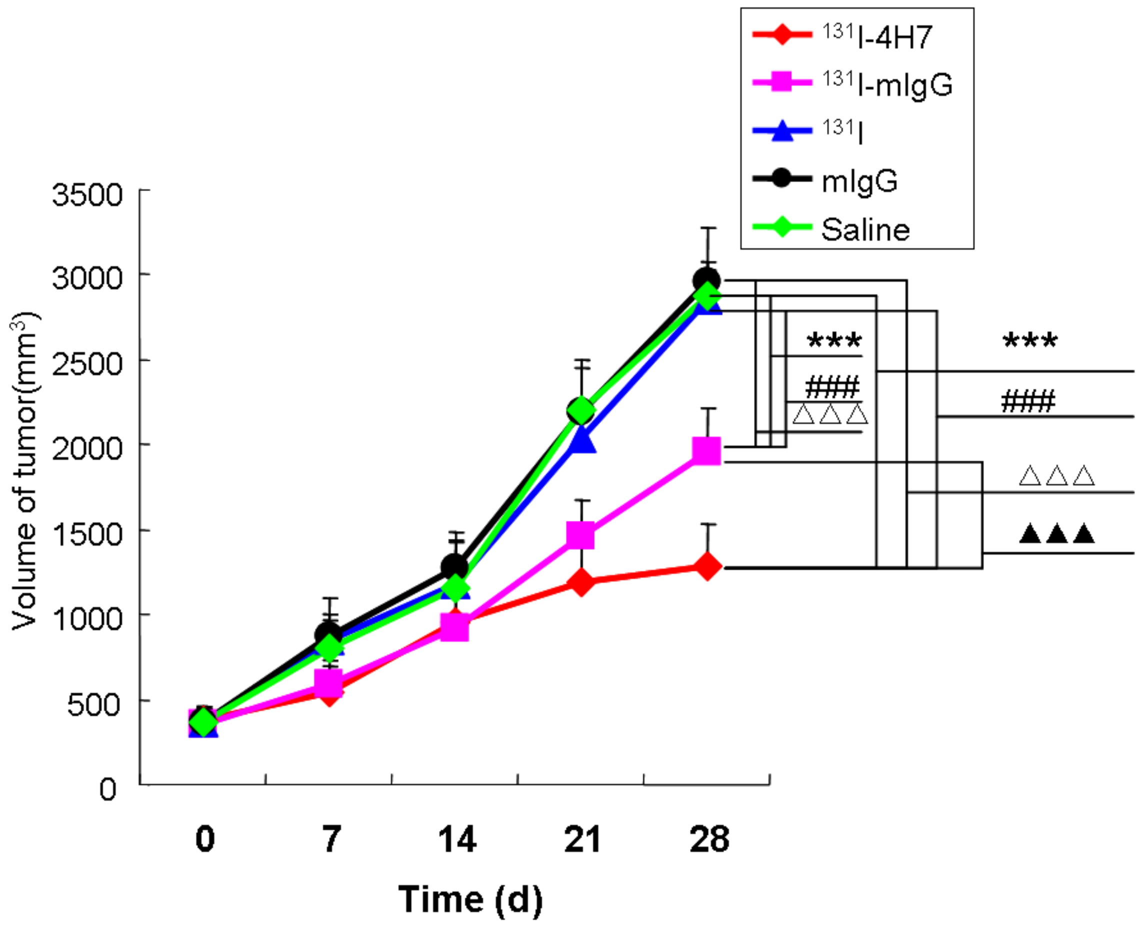

Supplementary Figure 4. The effect of drugs on tumor growth. Nude mice with renal cell carcinoma (RCC) xenografts were injected with saline, mIgG, ^{131}I, ^{131}I-mIgG, or ^{131}I-4H7. Tumor growth was monitored over 28 d. Data are mean ± SD, n = 8 (***: $p < 0.001$, Saline group vs. ^{131}I-mIgG and ^{131}I-4H7 group. $^{\triangle\triangle\triangle}$: $p < 0.001$, mIgG group vs. ^{131}I-mIgG and ^{131}I-4H7 group. $^{\#\#\#}$: $p < 0.001$, ^{131}I group vs. ^{131}I-mIgG and ^{131}I-4H7 group. $^{▲▲▲}$: $p < 0.001$, ^{131}I-mIgG group vs. ^{131}I-4H7 group).

Supplementary Table 1. Volume of tumor at day 0, 7, 14, and 21 among groups of saline, ^{131}I, ^{131}I-mIgG, and ^{131}I-4H7 according to the PET-CT results

	Day 0	Day 7	Day 14	Day 21
Saline (mm^3)	321.61±22.46	2367.14±416.69	3162.17±516.07	4305.38±454.63
^{131}I (mm^3)	324.52±23.54	2019.65±119.34	2642.29±176.29	4078.97±166.53
^{131}I-mIgG (mm^3)	315.58±18.17	1347.96±162.23$^{***, ###}$	1900.66±167.63$^{***, ###}$	2453.30±209.01$^{***, ###}$
^{131}I-4H7 (mm^3)	308.42±16.15	735.66±68.15$^{***, ###, △△△}$	1145.04±156.33$^{***, ###, △△△}$	1493.50±330.11$^{***, ###, △△}$

***: $p < 0.001$, ^{131}I, ^{131}I-mIgG, and ^{131}I-4H7 group vs. Saline group. $^{###}$: $p < 0.001$, ^{131}I-mIgG, and ^{131}I-4H7 group vs. ^{131}I group.$^{△△}$: $p < 0.01$; $^{△△△}$: $p < 0.001$, ^{131}I-4H7 group vs. ^{131}I-mIgG group.

Supplementary Table 2. Volume of tumor at day 0, 7, 14, 21, and 28 among groups of saline, ^{131}I, ^{131}I-mIgG, and ^{131}I-4H7 (Unit: mm^3)

Group	Day 0	Day 7	Day 14	Day 21	Day 28
Saline	361.61±81.60	806.19±160.06	1159.31±280.05	2202.20±290.58	2871.77±207.68
^{131}I	366.61±85.18	843.69±256.91	1178.06±250.27	2036.07±219.79	2846.77±184.90
^{131}I-mIgG	353.35±68.05	588.03±141.17$^{*, \#}$	924.37±182.29$^{\#}$	1466.15±209.74$^{***, \#\#\#}$	1955.03±260.48$^{***, \#\#\#}$
^{131}I-4H7	374.55±79.53	545.37±155.25$^{**, \#}$	953.80±213.33$^{\#}$	1190.35±221.00$^{***, \#\#\#, \triangle}$	1286.97±241.11$^{***, \#\#\#, \triangle\triangle\triangle}$

*: $p < 0.05$, **: $p < 0.01$, ***: $p < 0.001$, ^{131}I, ^{131}I-mIgG, and ^{131}I-4H7 group vs. Saline group. $^{\#}$: $p < 0.05$, $^{\#\#\#}$: $p < 0.001$, ^{131}I-mIgG, and ^{131}I-4H7 group vs. ^{131}I group. $^{\triangle}$: $p < 0.05$; $^{\triangle\triangle\triangle}$: $p < 0.001$, ^{131}I-4H7 group vs. ^{131}I-mIgG group.

Supplementary Table 3. Rate of tumor inhibition (%) at day 7, 14, 21, and 28 among groups of saline, ^{131}I, ^{131}I-mIgG, and ^{131}I-4H7

	Day 7	Day 14	Day 21	Day 28
^{131}I (%)	-4.65	-1.62	7.54	0.87
^{131}I-mIgG (%)	27.06	20.27	33.42	31.92
^{131}I-4H7 (%)	32.35	17.73	45.95	55.19

Supplementary Table 4. Description and comparison of biochemical characteristics between before and after different time-points of ^{131}I-4H7 administration

	before	2h	7d	14d	21d	28d
WBC(^10^9/L)	3.56±0.08	3.57±0.07	3.54±0.05	3.47±0.12	3.49±0.05	3.50±0.06
ALT (u/L)	37.20±2.17	35.8±2.86	35.40±2.61	35.00±2.24	35.80±2.28	35.80±2.86
AST (u/L)	29.20±2.28	28.80±1.92	27.60±2.07	27.40±1.67	27.60±2.70	27.80±2.28
BNU (mmol/L)	7.72±0.46	7.44±0.45	7.60±0.44	7.52±0.43	7.50±0.54	7.48±0.31
Cr (mmol/L)	68.80±2.28	67.60±3.51	67.00±4.47	71.00±2.83	66.80±4.82	67.40±3.85
